# Structural Characterization of Calcium Alginate Matrices by Means of Mechanical and Release Tests

**DOI:** 10.3390/molecules14083003

**Published:** 2009-08-12

**Authors:** Mario Grassi, Chiara Sandolo, Danilo Perin, Tommasina Coviello, Romano Lapasin, Gabriele Grassi

**Affiliations:** 1 Dipartimento di Ingegneria Chimica, dell'Ambiente e delle Materie prime, Università di Trieste, P.le Europa 1, 34127 Trieste, Italy; E-mail: romanol@dicamp.univ.trieste.it (R.L.); 2 Dipartimento di Chimica e Tecnologie del Farmaco, Università di Roma “La Sapienza”, P.le Aldo Moro 5, 00185 Roma, Italy; E-mails: chiara.sandolo@uniroma1.it (C.S.), tommasina.coviello@uniroma1.it (T.C.); 3 PROTOS Research Institute, Via Flavia 23/1, 34100 Trieste, Italy; E-mail: perin@protos-institute.org (D.P.); 4 Dipartimento di Scienze della vita, Università di Trieste, Via L. Giorgieri 1, I-34127 Trieste, Italy; E-mail: ggrassi@units.it (G.G.)

**Keywords:** calcium alginates, mechanical tests, diffusive tests, mesh size, equivalent network theory

## Abstract

In this paper we have concentrated on the characterization of calcium alginate hydrogels loaded with a model drug (myoglobin) by means of a mechanical approach; in addition, release tests of myoglobin from alginate hydrogels were performed. At a fixed temperature, relaxation tests (mechanical study) were carried out on matrices constituted by different polymer concentrations. The interpretation of the relaxation behavior of the different matrices was conducted using the generalized Maxwell model; as a result of this investigation it was possible to conclude that for polymer concentrations greater than 0.5 g/ 100 mL the matrices behaved as solid materials. In addition, it was observed that the mechanical properties of the matrices increased with polymer concentration. With regard to the release tests, the diffusion coefficient of myoglobin in the matrix in relation to polymer concentrations was determined. The mechanical and release data where then analyzed by Flory’s theory and by a modified free-volume theory, respectively, to estimate the network mesh size ξ. The comparison between the mesh sizes obtained by the two approaches showed a satisfactory agreement for polymer concentrations greater than 0.5 g/100 mL. It should be noted that the approach proposed here to determine the polymeric network meshes is absolutely general and can be advantageously applied to the characterization of other similar polymeric systems.

## 1. Introduction

Alginates, a family of natural polysaccharides, are widely used in industry and medicine for many applications, such as immobilization or isolation of chemicals and biological compounds in different encapsulation and cell entrapment systems [1,2]. Alginates are produced by the marine brown algae Macrocystis, Laminaria, Ascophyllum, Fucus, Eklonia and Pelvetia [3]. Microorganisms such as *Azotobacter vinelandii* and *Pseudomonas* also synthesize alginates, with different compositions [4]. Alginates can be regarded as a family of linear binary copolymers of 1,4-linked β-D-mannuronic acid (M) and α-L-guluronic acid (G) of varying composition and sequence. Mannuronic and guluronic acid residues are arranged in homopolimeric regions, blocks of guluronic (GGGG…) and mannuronic residues (MMMM...) and regions of alternating guluronic and mannuronic residues (GMGMGM…) [5,6]. The composition of alginate molecules, especially in terms of the structure of the blocks-regions, are dependent upon algal source and tissue and have an essential role in determining their gelling characteristics and functional properties as immobilization matrix [7,8]. In the presence of cations such as lead, copper, cadmium, barium, strontium, calcium and zinc, alginates produce thermally irreversible ionotropic gels; the selectivity for the binding of cations to alginates and the gel-forming properties vary with the composition and sequence of the polymer [8,9,10]. The gelling properties are also strongly affected by the polymer molecular weight as well as the molecular weight distribution; in addition, polymer concentration and conditions of gelation also influence the gelling properties.

Given the relevance of the polymeric network properties [11] for the proper design of polymer-based release systems, in this work we concentrated on the characterization of calcium alginates hydrogels by using mechanical and release tests carried out on matrices containing different polymer amounts. The mechanical characterization consisted of the relaxation experiments (normal stress relaxation at constant deformation) to determine 1) the hydrogel linear viscoelastic range and 2) to define the relaxation spectra and Young modulus by using the generalized Maxwell model [11]. On the basis of the Young modulus and of Flory’s theory [12], it was possible to determine the hydrogel cross-linking density ρ_x_. This value was then used to estimate the average polymeric mesh size ξ according to the equivalent network theory [13].

By the release tests, the delivery of a model drug (myoglobin) from cylindrical matrices characterized by different polymer concentrations was investigated. The interpretation (fitting) of the release data performed by a proper mathematical model based on the Fick’s law, allowed the determination of the dependence of myoglobin diffusion coefficient on polymer concentration. Subsequently, the analysis of diffusion coefficients by means of a modified free-volume theory [14,15] led to the estimation of the average polymeric mesh size ξ in relation to polymer concentration. The comparison of ξ values descending from mechanical and release tests evidenced a satisfactory agreement for polymer concentration greater than 0.5 g/100 mL. For lower values of polymer concentration, the determination of ξ via release tests became uncertain as evidenced by the considerable standard deviation associated to the mean value. It should be noted that the approaches suggested in this paper to determine the polymeric network meshes are absolutely general and they can be profitably applied to the characterization of other similar polymeric systems.

## 2. Results and Discussion

### 2.1. Mechanical characterization

Mechanical tests were performed on cylindrical gels (height ~ 1.9 cm; diameter 1.5 cm) at different deformation (ε_0_) (3%, 6%, 9%, 12%, 15%, 18%) in order to determine the linear viscoelasticity range. We found that, regardless of the polymer concentration *C*_p_ (= 0.5, 1.0, 2.5, 3.8, 5.0 g/100 mL, referred to as 0.5%, 1%, 2.5%, 3.8% and 5%, respectively), ε_0_ = 10% fall in the linear viscoelastic region. Thus, ε_0_ = 10% was selected as the constant deformation for the execution of the relaxation tests, which were aimed at the determination of gel Young modulus *E*. Being *E* time dependent for viscoelastic materials [16], its evaluation needs a proper interpretation of the relaxation data. In particular, assuming that the viscoelastic properties of the system can be conveniently described by the generalized Maxwell model [16], the dependence of the normal stress σ on time and constant deformation ε_0_ is given by [17]:

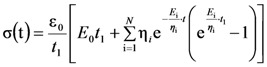
(1)
where *t* is time, η_i_ an *E*_i_ represent the generalized Maxwell model parameters, *N* is the number of Maxwell elements considered (apart from the pure elastic element characterized by *E*_0_) and *t*_1_ is the time required to get the desired deformation ε_0_. Obviously, when 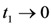 (instantaneous compression), Equation (1) becomes:

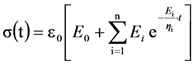
(2)
where η_i_/*E*_i_ can be seen as the relaxation times λ_i_. The Young modulus of the gel is given by the sum of the spring constants of all Maxwell elements (= 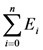). For gel systems that are cross-linked in solution and that did not undergo further swelling/shrinking before the mechanical test, Flory’s theory [12] establishes the following relation between the network crosslink density ρ_x_ and the Young modulus *E*:

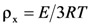
(3)
where *R* is the universal gas constant and *T* is the absolute temperature. The knowledge of the ρ_x_ value, together with the equivalent network theory [13], allow the estimation of the average mesh size (ξ) of the polymeric network. Indeed, the equivalent network theory starts from the evidence that, in the majority of the situations, a detailed description of a real polymeric network is very hard [18]. Therefore the equivalent network theory suggests to replace the real polymeric network by an idealized one, made up by a collection of spheres whose diameter coincides with the average network mesh size ξ (intended as the average distance between two consecutive network cross-links). Remembering the definition of cross-link density (moles of cross-links per hydrogel unit volume), it turns out that the volume of sphere is exactly equal to 1/(*N*_A_ ρ_x_) (this is the volume competing to each cross-link):

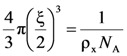
(4)
where *N*_A_ is the Avogadro number. Solving Equation (4) for ξ leads to:

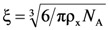
(5)
Equation (5) allows ξ estimation on the basis of ρ_x_ knowledge which in turn depends on the Young modulus (*E*) of the hydrogel, as determinable by the mechanical measurements.

**Figure 1:**
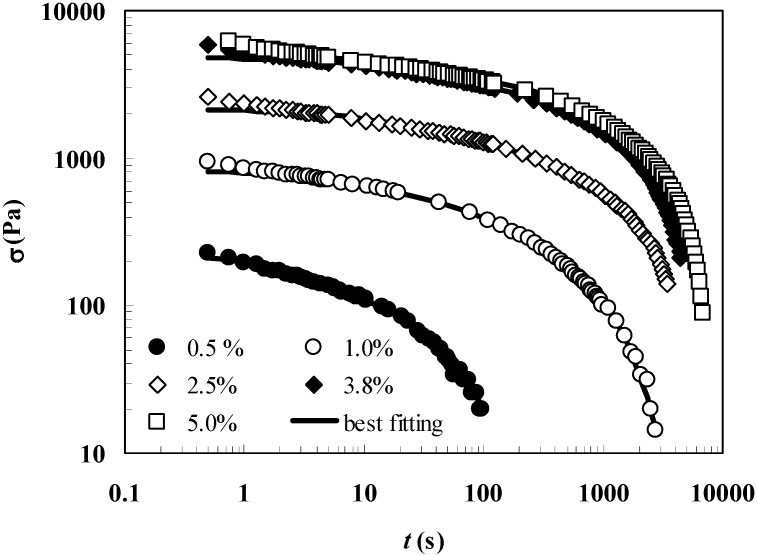
Comparison between the relaxation data (symbols) and model (Equation (1)) best fitting (solid line). σ and *t* represent the normal stress and time, respectively (constant deformation ε_0_ = 10%; *t*_1_ = 0.5 s). Different polymer concentrations are considered (0.5%, 1.0%, 2.5%, 3.8%, 5.0%).

Figure 1 shows, in a bi-logarithmic diagram, that the relaxation process (normal stress σ versus time *t*) progressively increases from low to high polymer concentrations. In particular, whereas for *C*_p_ = 0.5% the relaxation process lasts about 100 s, for *C*_p_ = 5.0% the relaxation process is approximately 100 times longer. In addition, Figure 1 reports the good agreement between the experimental relaxation data (symbols) referring to hydrogels characterized by different polymer concentrations (0.5%, 1.0%, 2.5%, 3.8%, 5.0%) and Equation (1) best fitting (solid line).

Following a consolidate way [16], Equation (1) fitting is carried out assuming that the relaxation times λ_i_ (= η_i_/*E*_i_) are not independent each other, but they are scaled by a factor of ten (λ_i_ = 10* λ_i-1_). Accordingly, only *E*_0_, η_i_ and λ_1_ represent the real fitting parameters of the model. The optimum number of Maxwell elements chosen to fit the experimental data is obtained minimizing the ratio between the sum of the squared errors and the number of the considered fitting parameters. The model fitting parameters and the derived parameters are reported in Table 1. It is evident that, with the exception of *C*_p_ = 2.5%, three Maxwell elements are required to satisfactory fit (see *F* vales) the experimental data. The presented data also indicate that no pure elastic components (*E*_0_ = 0) are present and that the Young modulus *E* together with the crosslink density ρ_x_, considerably increases with the polymer concentration. Although the *C*_p_ = 2.5% case requires the presence of a pure elastic component, this leading to consider four Maxwell elements, its contribution to the overall mechanical behavior is very limited representing only the 3% of *E*.

Table 1Model best fitting parameters (λ_1_, η_1_, η_2_, η_3_, *E*_0_) and derived parameters (*E*_1_, *E*_2_, *E*_3_, *E*, ρ_x_, ξ) referring to relaxation data. *t*_1_ is the time required to get the desired cylindrical gel deformation (ε_0_ = 10%) while *F* indicates “*F-statistic*” parameter. Values are reported as mean ± standard deviation.

**Table:** 

***C*_p_(w/v)**	**0.5**	**1.0**	**2.5**	**3.8**	**5.0**
**Fitting parameters**					
***F*(ν_1_, ν_2_, 0.95)**	*F*(1, 181, 0.95) < 11365	*F*(3, 311, 0.95) < 53299	*F*(4, 676, 0.95) < 261761	*F* (3, 877, 0.95) < 907204	*F*(3, 858, 0.95) < 50660
***t*_1_(s)**	0.5	0.5	0.5	0.5	0.75
**λ_1_(s)**	3.3 ± 0.2	8.9 ± 0.1	15.0 ± 0.2	20.1 ± 0.1	22.5 ± 0.1
**η_1_(Pa s)**	2745 ± 207	16380 ± 943	76638 ± 1565	214758 ± 2724	303546 ± 11285
**η_2_(Pa s)**	38297 ± 1491	275052 ± 5603	853343 ± 14609	246630 ± 11870	1904260 ± 79867
**η_3_(Pa s)**	68038 ± 2693	2890350 ± 8136	1484800 ± 219043	49574000 ± 23398	65121400 ± 263613
***E*_0_(Pa)**	0	0	688 ± 49	0	0
**Derived parameters**					
***E*_1_ = η_1_/λ_1_**	818 ± 72	1835 ± 106	5088 ± 127	10677 ± 136	13545 ± 508
*E*_2_ = η_2_/(10*λ_1_)	1141 ± 45	3082 ± 63	5665 ± 87	12262 ± 59	8497 ± 356
*E*_3_ = η_2_/(100*λ_1_)	203 ± 8	3239 ± 9	9858 ± 145	24647 ± 11	29059 ± 117
***E* = Σ_i_*E*_i_**	2162 ± 85	8158 ± 124	21294 ± 124	47587 ± 149	511902 ± 631
**ρ_x_** **(mol cm^-3^)**	(0.29 ± 0.01)*10^-6^	(1.10± 0.01)*10^-6^	(2.80± 0.03)*10^-6^	(6.4 ± 0.02)*10^-6^	(6.9 ± 0.1)*10^-6^
**ξ(nm)**	22.0 ± 0.3	14.0 ± 0.1	10.0 ± 0.1	7.9 ± 0.01	7.7 ± 0.03

**Table:** 

***C*_p_(w/v)**	**3.8**	**5.0**
**Fitting parameters**		
***F*(ν_1_, ν_2_, 0.95)**	*F*(3, 877, 0.95) < 907204	*F*(3, 858, 0.95) < 50660
***t*_1_(s)**	0.5	0.75
**λ_1_(s)**	20.1 ± 0.1	22.5 ± 0.1
**η_1_(Pa s)**	214758 ± 2724	303546 ± 11285
**η_2_(Pa s)**	246630 ± 11870	1904260 ± 79867
**η_3_(Pa s)**	49574000 ± 23398	65121400 ± 263613
***E*_0_(Pa)**	0	0
**Derived parameters**		
***E*_1_ = η_1_/λ_1_**	10677 ± 136	13545 ± 508
*E*_2_ = η_2_/(10*λ_1_)	12262 ± 59	8497 ± 356
*E*_3_ = η_2_/(100*λ_1_)	24647 ± 11	29059 ± 117
***E* = Σ_i_*E*_i_**	47587 ± 149	511902 ± 631
**ρ_x_** ** (mol cm^-3^)**	(6.4 ± 0.02)*10^-6^	(6.9 ± 0.1)*10^-6^
**ξ(nm)**	7.9 ± 0.01	7.7 ± 0.03

**Figure 2:**
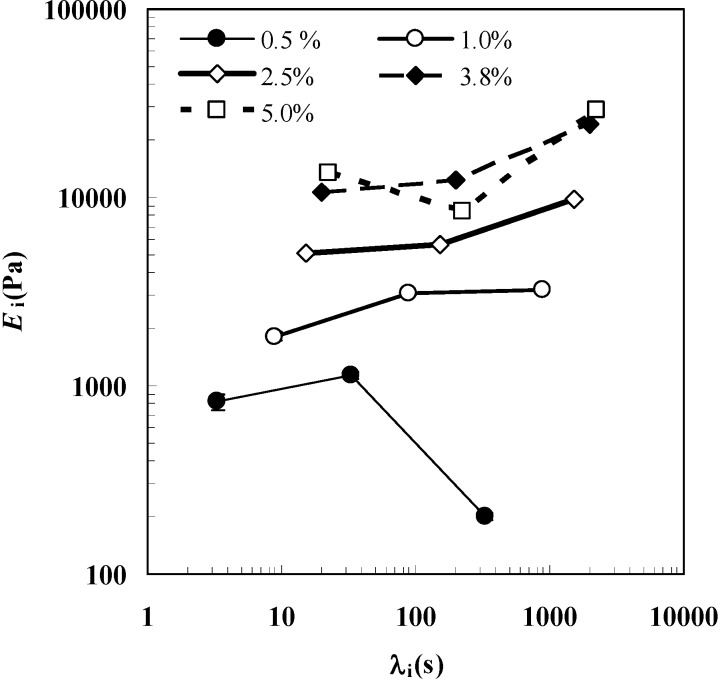
Relaxation spectra referring to *C*_p_ = 0.5%, 1.0%, 2.5%, 3.8% and 5.0% hydrogels. This picture relies on the data shown in Table 1.

Interestingly, Figure 2, reporting the relaxation spectra (*E*_i_ vs λ_i_) referring to the five polymer concentrations considered in this work, clearly shows that hydrogels characterized by *C*_p_ > 0.5 show a typical solid-like behavior as the relevance (i.e. weight) of longer relaxation times is prevalent on the smaller ones. In other words, the *E*_i_ vs λ_i_ curve has a positive slope. In addition, the increase of *C*_p_ determines a rise of both *E*_i_ and λ_1_ (relaxation process becomes slower and slower). It is worth noticing that gel properties, regardless of the polymer concentration, are not significantly modified in the presence of our model drug (myoglobin; van der Waals radius 2.1 nm [19]) up to the studied concentration (7.6 mg/cm^3^).

As stated above, the mechanical characterization can also provide information about the gel nano-structure. Indeed, Equation (3) allows the estimation of the cross-link density ρ_x_ of the polymeric network. In turn, the equivalent network theory [13] allows, via Equation (5), the determination of the gel average mesh size ξ. Table 1 indicates that ξ decreases in a not linear manner, with the polymer concentration from about 22 nm (*C*_p_ = 0.5%) to 7.7 nm (*C*_p_ = 5.0%). In this last case, the mesh dimension is less than two times the van der Waals diameter of myoglobin (4.2 nm [19]); this indicates that the polymeric network should exert a considerable reduction of model drug mobility.

### 2.2. Release characterization

The mesh size (ξ) of the polymeric network was also estimated from the drug (myoglobin) diffusion coefficient in the polymeric network (*D*) and in the swelling agent (water in our case, *D*_0_). Indeed, it is well known that the knowledge of the drug diffusion coefficient in the polymeric network (*D*) and in the swelling agent (water in our case, *D*_0_) allows an approximate estimation of the polymeric network average mesh size ξ [14,15]. Peppas and coworkers [14,15] combining the free volume theory [20] with the assumption that the probability that a solute of radius *r*_s_ has to pass through an opening of diameter ξ is linearly dependent on the ratio 2*r_s_*/ξ, determined the following relation between the ratios *D*/*D*_0_, 2*r_s_*/ξ and polymer volume fraction:

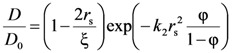
(6)
where *k*_2_ is a model parameter to be determined. Although the authors suggest that the product *k*_2
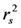
_ can be approximately set equal to one, we preferred to estimate it from the experimental data. The real difficulty in *k*_2_ determination comes from the fact that Peppas and co-workers did not provide a functional dependence of ξ on polymer volume fraction φ (if the function ξ(φ) were known, *k*_2_ could be simply determined by fitting Equation (6) on experimental data of *D*/*D*_0_ vs φ). Accordingly, *k*_2_ was determined observing that in the limit φ → 0, ξ(φ) becomes infinite, *r*_s_/ξ(φ) becomes zero and the exponential term in Equation (6) can be transformed in the light of Taylor series development (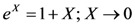) as *X* = (φ/(1-φ)) becomes zero:

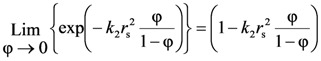
(7)

Thus, in the limit φ → 0, we have:

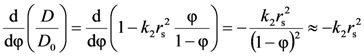
(8)

Accordingly, *k*_2_ can be determined as:

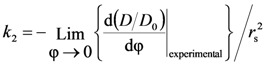
(9)

The determination of myglobin diffusion coefficient inside the gel network can be performed recurring to a mathematical model based on the Fick law of diffusion. Due to gel symmetry, the intrinsically three dimensional diffusive problem could be reduced to a simpler two dimensional one:

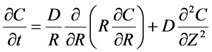
(10)
where *D* is the drug diffusion coefficient in the gel, *t* is time, *C* is the drug concentration (mass/volume) in the cylinder, *R* and *Z* are the radial and axial axes, respectively. This equation must satisfy the following initial and boundary conditions:

Initial conditions:

*C*(*Z*, *R*) = *C*_0_            -*Z*_c_ ≤ *Z* ≤ *Z*_c_ 0 ≤ *R* ≤ *R*_c_ (11)

*C*_r_ = 0 (12)

Boundary conditions

*C*(*Z*, *R*_c_, *t*) = *C*(±*Z*_c_, *R*, *t*) = *k*_p _*C*_r_(*t*) (13)

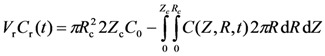
(14)
where 2*Z*_c_ and *R*_c_ are, respectively, cylinder height and radius, *C*_0_ is the initial drug concentration in the cylinder, *C*_r_ and *V*_r_ are the drug concentration and the volume of the release medium while *k*_p_ is the drug partition coefficient between the cylindrical gel and the environmental release fluid. Equations (11)–(12) state, respectively, that the gel is uniformly loaded with a drug at *C*_0_ concentration, while the release environment is initially drug free. Equation (13) expresses the partitioning condition at the cylinder/release fluid interface, while Equation (14) is a drug mass balance for the gel/release fluid system allowing to state the relation between *C*_r_ and *C*(*Z*, *R, t*).

From the experimental point of view, myoglobin release was studied considering cylindrical gels (height = 1.9 cm; diameter = 1.5 cm) characterized by different polymer concentration *C*_p_ (= 0.5, 1.0, 2.0, 3.0, 3.5, 4.0, 4.5, 5.0 and 6.5%). Although our gels did not undergo further swelling during the release tests (no dimensional increases were observed), they showed an erosion whose magnitude was proportional to the time but almost independent from the polymer concentration (after 8 hours, about 15% of the whole polymeric content was found in the release environment). As gel shape and dimensions were not modified during the whole release period (8 hours), we concluded that gel erosion was not superficial but “bulk” and thus drug release was always imputable to a diffusive mechanism. On the contrary, the observed erosion should, in principle, increase myoglobin mobility inside the gel and, at the same time, improve the resistance effect exerted by the hydrodynamic boundary layer surrounding the gel matrix. Indeed, the presence of a sustaining net (to suspend the gel in the release environment) enveloping the gel matrix should favor the polymer entrapment and residence at the gel release environment interface. In order to account for this complex situation in a simple manner, we supposed that myoglobin diffusion coefficient was time dependent according to:

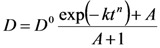
(15)
where *D*^0^ is the value of the myoglobin diffusion coefficient at the beginning (time *t* = 0; i.e when the gel is unaffected by erosion) while *k*, *A* and *n* are fitting parameters. This equation states that *D* modifies with time according to a stretched exponential law. Thus, the entire mathematical model is represented by Equations (10)–(15) and Equation (15) was introduced for the *D*^0^ estimation. *D*^0^ value, independent on the erosion effect, was used to estimate the network mesh size by Equation (6). In order to reduce model fitting parameters to 3 (*D*^0^, *k*, *A*), the partition coefficient *k*_p_ was set equal to 1 and *n* was set equal to 6 (this corresponds to a sharp *D* variation from *D*^0^ to *D*(*t* = ∞)). The numerical solution of the model was performed according to the control volume method [21]. This approach is based on the subdivision of the calculation domain into a number of non-overlapping control volumes (rings, in our 2D frame) where Equation (10) is integrated assuming uniform model drug concentration in each control volume. In order to ensure the reliability of the numerical solution, the domain was subdivided into 100 control volumes in the radial and axial direction (for a total of 10^4^ control volumes) and the integration time step was set equal to 22.5 s.

**Figure 3:**
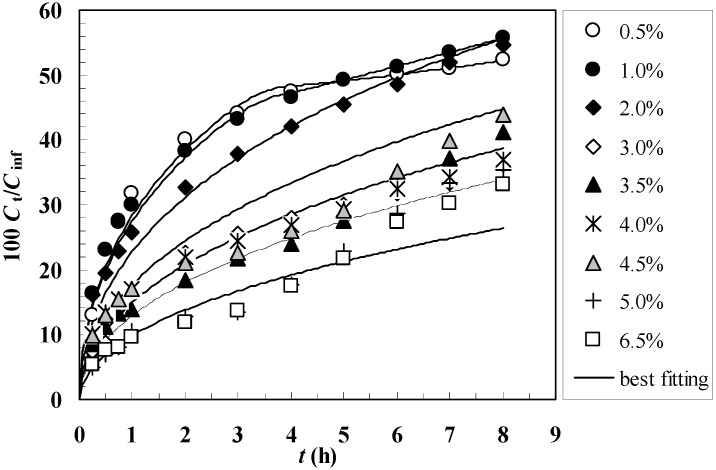
Comparison between model best fitting (solid line) and experimental release data (symbols) referring to gels characterized by different polymer concentrations *C*_p_ (0.5% - 6.5%). *C*_t_ and *C*_inf_ indicate the drug concentration in the release environment after time *t* and after a very long time (infinite), respectively.

Figure 3 shows the comparison between the model best fitting (solid line) and the experimental release data (symbols) referring to gels characterized by different polymer concentrations *C*_p_. It can be seen that the agreement between the experimental data and the model best fitting is satisfactory, as also witnessed by the *F* statistic values reported in Table 2. Limited to the *C*_p_ = 0.5 and 1.0% cases, it is necessary to adopt the time dependent diffusion coefficient defined by Equation (15). In all the other situations, the statistic indicates that the use of Equation (15) is unnecessary so that the model fitting parameters reduce to *D*^0^ = *D*. This means that for *C*_p_ > 1, in the light of the “high” polymer concentration, the effect of the boundary layer and the reduction of gel network connectivity do not sensibly affect the average mobility of myoglobin (and, thus, its release rate). On the contrary, for lower polymer concentrations, this effect becomes important. Indeed, as the extent and nature (presence of eroded polymer) of the boundary layer mainly depends on the conditions of the hydrodynamic environment and on the net presence (conditions equal for all our gels), it seems reasonable that the combined effect of bulk erosion and boundary layer build up is more evident for lower polymer concentrations. The fact that for *C*_p_ = 0.5 and 1.0% the model fitting leads to a decrease of *D* (*k* > 0 in Equation (15)), indicates that the permeability of the boundary layer to myoglobin reduces with time; this could imply a boundary layer thickness increase and/or the increase of eroded polymer concentration in it. The model best fitting reveals that the increase of *C*_p_ from 0.5% to 6.5% results in a decrease of the myoglobin diffusion coefficient of about one order of magnitude (see Table 2).

**Table 2:** Model best fitting parameters (*D*^0^, *k*, *A*) and derived parameters (ξ, *D*^0^/*D*_0_, *k*_2_) referred to the release data. *D*_0_ is the diffusion coefficient of myoglobin in water (37°C) [11], *n* (= 6) is Equation (15) exponent while *F* indicates “*F-statistic*” parameter. Values are reported as mean ± standard deviation.

***C*_p_(w/v)**	**0.5**	**1.0**	**2.0**
***F*(ν_1_, ν_2, _0.95)**	*F*(2, 10, 0.95) < 324	*F*(2, 10, 0.95) < 664	*F*(1, 10, 0.95) < 305
***D*^0^(cm^2^/s)**	(1.5 ± 0.1)*10^-6^	(1.4 ± 0.05)*10^-6^	(0.94 ± 0.04)*10^-6^
***k*(s^-6^)**	(6 ± 3)*10^-25^	(6 ± 2)*10^-25^	-
***A*(-)**	(8 ± 3)*10^-3^	(27 ± 3)*10^-3^	-
***k*_2_(nm^-2^)**	2.96 ± 0.22	2.96 ± 0.22	2.96 ± 0.22
100*D^0^/*D*_0_(-)	96 ± 6.5	90 ± 3	60 ± 2
**ξ(nm)**	59 ± 53	25.7 ± 4.4	8.7 ± 0.4
***C*_p_(w/v)**	**3.0**	**3.5**	**4.0**
***F*(ν_1_, ν_2, _0.95)**	*F*(1, 10, 0.95) < 1013	*F*(2, 10, 0.95) < 191	*F*(1, 10, 0.95) < 305
***D*^0^(cm^2^/s)**	(0.38 ± 0.10)*10^-6^	(0.28 ± 0.05)*10^-6^	(0.38 ± 0.03)*10^-6^
***k*(s^-6^)**	-	-	-
***A*(-)**	-	-	-
***k*_2_(nm^-2^)**	2.96 ± 0.22	2.96 ± 0.22	2.96 ± 0.22
100*D^0^/*D*_0_(-)	24 ± 1	18.0 ± 0.3	24 ± 2
**ξ(nm)**	5.20 ± 0.04	4.90 ± 0.02	5.1 ± 0.1
***C*_p_(w/v)**	**4.5**	**5.0**	**6.5**
***F*(ν_1_, ν_2, _0.95)**	*F*(1, 10, 0.95) < 51.5	*F*(1, 10, 0.95) < 155	*F*(1, 10, 0.95) < 87
***D*^0^(cm^2^/s)**	(0.55 ± 0.04)*10^-6^	(0.16 ± 0.006)*10^-6^	(0.22 ± 0.02)*10^-6^
***k*(s^-6^)**	-	-	-
***A*(-)**	-	-	-
***k*_2_(nm^-2^)**	2.96 ± 0.22	2.96 ± 0.22	2.96 ± 0.22
100*D^0^/*D*_0_(-)	35 ± 3	10 ± 0.4	14 ± 1.2
**ξ(nm)**	5.6 ± 0.2	4.5 ± 0.01	4.6 ± 0.04

Remembering that the following relation between polymer concentration *C*_p_ and polymer volume fractions φ holds:

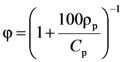
(16)
where ρ_p_ is polymer density (1.8 g/cm^3^ [22]), it is possible estimating the dependence of myoglobin diffusion coefficient on j. This, in turn, allows the estimation of *k*_2_ (see Equation (6)) according to Equation (9):

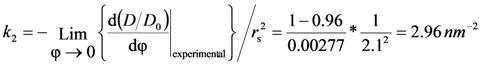
(9’)

On the basis of this result, Equation (6) can be used to estimate the average mesh size of the polymeric network and its standard deviation σ_ξ_ (calculated according to the error propagation law):

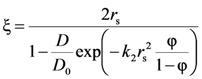
(6’)

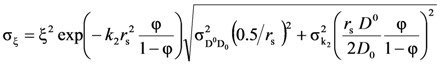
(17)
where 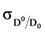 and 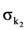 are, respectively, the standard deviation of the *D*^0^/*D*_0_ ratio and of *k*_2_ (see Table 2). Table 2 shows that ξ reduces with *C*_p_ in a non-linear manner and that its standard deviation is considerably high for *C*_p_ = 0.5%. This underlines the criticality of ξ determination for very low values of polymer volume fraction. In this regards it is worth mentioning that the most important contribute to 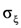 is given by the uncertainty related to the estimation of *D*^0^/*D*_0_, i.e. 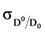.

**Figure 4:**
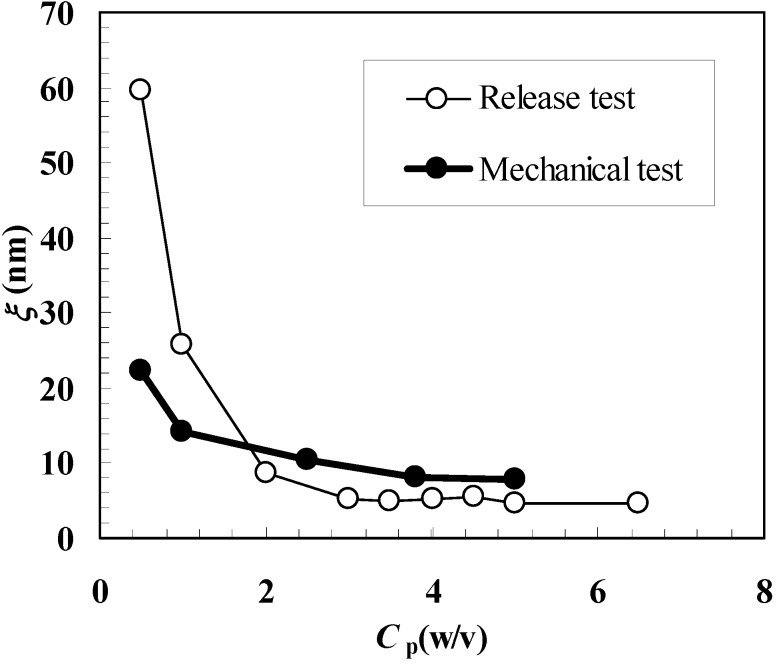
Comparison between the values of the polymeric network average mesh size (ξ) calculated according to the mechanical and release approaches. *C*_p_ represents the polymer concentration.

Figure 4 shows the comparison between ξ estimation according to the mechanical and release approaches. It can be seen that, with the exception of *C*_p_ = 0.5%, similar results are obtained even if the release approach yields higher ξ values for *C*_p_ = 1.0% and smaller values for *C*_p_ ≥ 2.0%. These differences cannot be imputed to the different temperatures at which mechanical (25 °C) and release (37 °C) tests were performed. The high standard deviation associated to ξ for *C*_p_ = 0.5% suggests a low reliability of the release approach for what concerns ξ estimation at very low *C*_p_ (or j) values. This is reasonable as, in this φ range, the effect of polymeric network on model drug diffusion (mobility) is very low and a reliable experimental determination of the diffusion coefficient becomes problematic. Probably, in this diluted range, it would be necessary to consider bigger model drugs whose van der Waals diameter is not negligible in comparison to ξ (myglobin’s van der Waals diameter is approximately 1/5 and 1/10 of ξ calculated according to the mechanical and release approach, see Table 1 and Table 2.). On the contrary, the mechanical approach seems to be more reliable (at least judging from the standard deviation values associated to ξ, see Table 1) also in this diluted range. Again, the reason for this relies on the gel characteristic tested by the mechanical approach, i.e. network connectivity that, although low, is able to confer to the water-polymer system a mechanical behavior more similar to that of a solid rather than to that of a solution. Anyway, diluted range apart, Equation (6) proved to be a reliable tool for ξ estimation.

## 3. Experimental Section

### 3.1. Materials

Alginate (guluronic acid content 65 ÷ 75%; mannuronic acid content 35 ÷ 25%; Lot. 3-0421E; Viscosity 200-400 mPas for a 1% solution, at 20 °C) was provided by Carbomer (USA). Horse heart myoglobin (molecular weight 17,800) was purchased from Fluka (Germany). All the other products and reagents were of analytical grade. Distilled water was used in all experiments.

### 3.2. Hydrogel preparation

Alginate has been purified dissolving the polymer (5 g) in distilled water (1 L) followed by homogenization *via* magnetic and mechanical stirring at room temperature for 24 hours. The solution was exhaustively dialyzed (Visking Tubing, cut-off 12,000 ÷ 14,000) at 4 °C against distilled water to remove the excess of ions. Dialysis ended when the conductivity of the washing water was approximately equal to that of pure water, ≈ 1 μS at 25 °C. The dialyzed solution was then neutralized by the addition of 0.2 N NaOH. In this manner, the alginic acid, derived from the prolonged dialysis, was transformed into its sodium salt (sodium alginate). Finally, the sample was freeze-dried and stored in sealed vessels in the presence of CaCl_2_ as drying agent. Drug loaded hydrogels were obtained by adding a known amount of myoglobin to distilled water, followed by addition of a calculated amount of purified alginate. System homogenization was ensured by 24 hours magnetic stirring. Alginate solution was poured into a glass tube (height 1.9 cm, diameter 1.5 cm) sealed at top and bottom by dialysis membranes (cut-off 12,000 ÷ 14,000). The sealed glass tube was put in a CaCl_2_ (0.05 M) and NaCl (0.4 M) aqueous solution for three days in order to get a homogeneous cross-linking of the alginate chains [23]. In the light of this gel preparation procedure, no significant gel shrinking or swelling after crosslinking were detected. Thus, polymer concentration in the gel coincided with that of the original aqueous alginate solution. Myoglobin free hydrogels were obtained according to the same procedure except for drug absence in the initial aqueous alginate solution. Final polymer concentrations (*C*_p_) spanned from 0.5 to 6.5 g/100 cm^3^ (in the text also referred to as 0.5% - 6.5%) while myoglobion concentration was 7.6 mg/cm^3^.

### 3.3. Mechanical tests

A software-controlled dynamometer, TA-XT2i Texture Analyzer (Stable Micro Systems, UK), with a 5 Kg load cell, a force measurements accuracy of 0.0025 % and a distance resolution of 0.0025 mm (according to the instrument specifications), was used for the mechanical characterization of the gel samples. The hydrogel stress relaxation [σ(t)] at constant deformation (ε_0_) was measured. The pre-test speed was set up at 2.0 mm/s while test speed was 4.0 mm/s. The probe used was an aluminium cylinder with a diameter of 35 mm (P35). The study was carried out at 25°C, with a controlled temperature bath (± 0.1 °C) (Haake, model DC 10). In order to determine the linear viscoelasticity range, different deformations were studied (3%, 6%, 9%, 12%, 15%, 18%). As ε_0_ = 10% falls within the linear viscoelasticity range for all the polymer concentrations considered, ε_0_ = 10% was assumed for the relaxation study (up to 8,000 s).

### 3.4. Release tests

During the release experiment, carried out in distilled water (200 mL) at 37 °C, the cylindrical hydrogel was kept at a certain height from the bottom of the vessel containing the release environment by a thin web, while the medium was gently magnetically stirred. Three mL samples were withdrawn from the solution at appropriate time intervals and replaced by the same amount of fresh solvent (concentration data were corrected for dilution). Myoglobin was spectrophotometrically detected at 409 nm (Perkin-Elmer, lambda 3a, UV-Visible spectrometer) using quartz cells with path-lengths of 1.0 cm. All experiments were carried out in triplicate and the values reported in the present paper represent mean values and lay within 10% of the mean. Hydrogel erosion, in terms of polymer dissolution in the medium during the release experiments, was quantitatively determined by a colorimetric method [24] using phenol in the presence of sulphuric acid. Obtained results indicate that polymer erosion is about 15% in the first 8 h, regardless the initial polymer concentration.

## 4. Conclusions

The mechanical tests (relaxation at constant deformation) performed on alginate hydrogels revealed that the linear viscoelastic range depends on polymer concentration. However, deformations ≤ 10% fall in the linear viscoelastic range regardless of the polymer concentration (in the studied range). In addition, relaxation tests prolonged up to 8000 s showed that for polymer concentration ≥ 1%, alginate hydrogel is characterized by a typical solid-like behavior while for the lowest polymer concentration considered (0.5%) a behavior approaching the liquid-like one was observed. The release tests revealed that the model drug (myoglobin) diffusion coefficient *D*^0^ strongly depends on the polymer concentration (approximately one order of magnitude decrease was observed moving from *C*_p_ = 0.5 to 6.5%). In addition, the mathematical model developed to measure *D*^0^ proved that the effect of the hydrogel bulk erosion (15% after 8 hours regardless of the polymer concentration) and the formation of the surrounding hydrodynamic boundary layer played a relevant role only for the lowest polymer concentrations studied (0.5% and 1.0%).

The mechanical and release tests allowed the determination of polymeric network average mesh size ξ. The comparison of the results descending from the two approaches underlined a substantial agreement except for the lowest polymer concentration studied (0.5%) where the release approach led to a higher estimation. The high standard deviation associated to this value led to the conclusion that in a very dilute polymer concentration range, the determination of *D*^0^ becomes problematic and the mechanical approach seems to be more reliable.
